# New Diglyme‐based Gel Polymer Electrolytes for Na‐based Energy Storage Devices

**DOI:** 10.1002/cssc.202101445

**Published:** 2021-09-30

**Authors:** Binson Babu, Marcel Enke, Sofiia Prykhodska, Alexandra Lex‐Balducci, Ulrich S. Schubert, Andrea Balducci

**Affiliations:** ^1^ Institute for Technical Chemistry and Environmental Chemistry Friedrich Schiller University Jena Philosophenweg 7a 07743 Jena Germany; ^2^ Laboratory of Organic and Macromolecular Chemistry (IOMC) Friedrich Schiller University Jena Humboldtstr. 10 07743 Jena Germany; ^3^ Center for Energy and Environmental Chemistry Jena (CEEC Jena) Friedrich Schiller University Jena Philosophenweg 7a 07743 Jena Germany

**Keywords:** batteries, diglyme, gel-polymer electrolytes, sodium, supercapacitors

## Abstract

This work presents for the first time a new diglyme‐based gel polymer (DOBn‐GPE) suitable for Na‐based energy storage devices. The DOBn‐GPE, which contains a methacrylate‐based polymer, exhibited an excellent high ionic conductivity (2.3 mS cm^−1^ at 20 °C), broad electrochemical stability (>5.0 V), and high mechanical stability. DOBn‐GPE could be successfully used for the realization of Na‐ion capacitors, sodium‐metal batteries, and sodium‐ion batteries, displaying performance comparable with those of systems containing liquid electrolytes at room temperature and at 60 °C. The results of these investigation indicated that the development of diglyme‐based gel polymer electrolytes represents a promising strategy for the realization of advanced Na‐based energy storage devices.

## Graphical abstract

**Na‐based energy storage**: The new diglyme‐based gel polymer electrolyte (DOBn‐GPE), consisting of methacrylate‐based polymer, exhibits high ionic conductivity, broad electrochemical stability, and high mechanical stability. The utilization of DOBn‐GPE with graphite shows comparable performance to that of liquid electrolyte. The successful utilization of DOBn‐GPE for the realization of Na‐ion capacitors, sodium‐metal batteries, and sodium‐ion batteries is demonstrated at room temperature and at 60 °C.

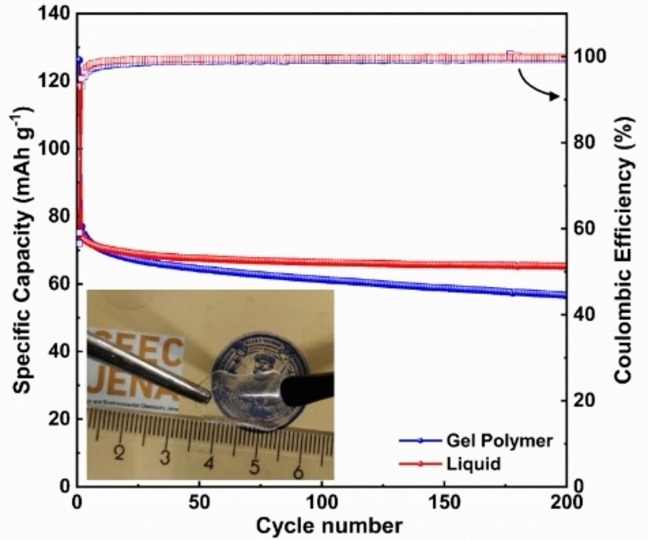

## Introduction

In the last decade, due to increasing concerns about the long‐term availability of lithium,^[1]^ several efforts have been dedicated towards the development of energy storage devices that provide an alternative to lithium‐ion batteries (LIBs). Among them, the systems based on sodium (Na) are nowadays considered the most interesting. Sodium is very abundant, widely distributed around the world, cheaper compared to lithium, and the performance of Na‐based energy storage devices is approaching that of LIBs.^[2]^

In the last years, practically all classes of materials utilized and investigated for LIBs have been also considered for Na‐based devices. In many cases the “transition” from lithium to sodium is rather straightforward, but there are also materials utilized in LIBs that cannot be easily implemented in Na‐based systems. This is the case with graphite, which is the state‐of‐the art anode of commercial LIBs and, also, of commercial Li‐ion capacitors (LICs).^[3]^ Graphite is known to be an excellent host structure, in which a large variety of cations and anions can be reversibly inserted/extracted.^[4]^ Nevertheless, due to the thermodynamic instability of Na‐rich binary graphite intercalation compounds (b‐GICs) formed between the graphene layers, the insertion of Na into graphite has been a challenge for a long time, and this anodic material could not be conveniently used in Na‐based devices.^[5]^ In 2014, Jache and Adelhelm^[6]^ and Kim et al.^[7]^ demonstrated that the use of ether (glymes)‐based electrolytes allows the formation of ternary graphite intercalation compound (t‐GICs), which can be reversibly intercalated/de‐intercalated to/from the graphite electrodes.^[8]^ Afterward, several studies showed the successful utilization of diglyme‐based electrolytes in Na‐ion batteries (NIBs)^[9]^ and Na‐ion capacitors (NICs).^[10]^ Moreover, Seh et. al.^[11]^ reported that the use of a diglyme‐based electrolyte significantly inhibits the dendrite formation due to the formation of a uniform inorganic solid electrolyte interphase (SEI) layer on the metal surface, which enables the long‐term highly reversible plating/stripping in Na‐metal anodes. This facilitates the safe long‐term usage of metallic sodium in high‐energy rechargeable sodium‐metal batteries (NMBs).^[12]^

Currently, liquids electrolytes based on mixtures of organic solvents are mainly used for the realization of Na‐based systems.^[13]^ Nevertheless, the flammability and volatility of these electrolytes is limiting the overall safety of these devices and, consequently, the introduction of alternative and safer electrolytes is presently considered as an aspect of great importance. Among the various alternatives, gel‐polymer electrolytes (GPEs) are one of the most promising.^[14]^ GPEs, which consist of a polymeric matrix soaked with a liquid electrolyte (LE), display good ionic conductivity at room temperature (≈10^−3^ S cm^−1^) and high mechanical stability. Their use improves the safety of the devices by inhibiting the dendrite growth and by avoiding leakage of electrolytes, and also provides sufficient mechanical stability to develop flexible energy storage devices.[^14a^, ^15^]

So far, only a limited number of GPEs have been considered for Na‐based energy storage systems. Among them, poly(methyl methacrylate) (PMMA),^[16]^ poly(ethylene oxide) PEO, and poly(vinylidene fluoride‐*co*‐hexafluoropropylene) (PVdF‐HFP)[^14b^, ^17^] with carbonate‐based solvents are the most studied ones.^[18]^ On the other hand, only Zhang et al.^[19]^ considered the development of GPEs containing ether‐based solvents. The authors reported a polysulfonamide‐supported poly(ethylene glycol) divinyl ether‐based polymer electrolyte and demonstrated that this GPEs display high ionic conductivity at ambient temperature (1.2×10^−3^ S cm^−1^), a wide electrochemical stability, and can be successfully utilized in NIBs. Taking into account these promising results, and the fact that the use of these glyme‐based electrolytes allows the use of graphite in Na‐based systems, further studies about this type of GPE appear to be of high interest.

In this study, we report the investigation of a new diglyme‐based GPE (DOBn‐GPE) suitable for Na‐based energy storage devices. The DOBn‐GPE contains a methacrylate‐based polymer, which has already been successfully used in combination with the conventional LP30 as electrolyte for LIBs and LICs.^[20]^ First, the physiochemical properties of the new DOBn‐GPE are determined in detail and compared with those of the liquid electrolyte soaked in the polymer matrix (1 m NaPF_6_ in diglyme). Subsequently, the use of DOBn‐GPE in NICs, NMBs, and NIBs containing graphite, prussian blue (PB), activated carbon (AC), and metallic sodium as electrode materials is investigated in detail, and these results are compared with those obtained with the LE at room temperature and at 60 °C.

## Results and Discussion

### Synthesis and characterization of diglyme‐based gel GPEs

**Figure 1:**
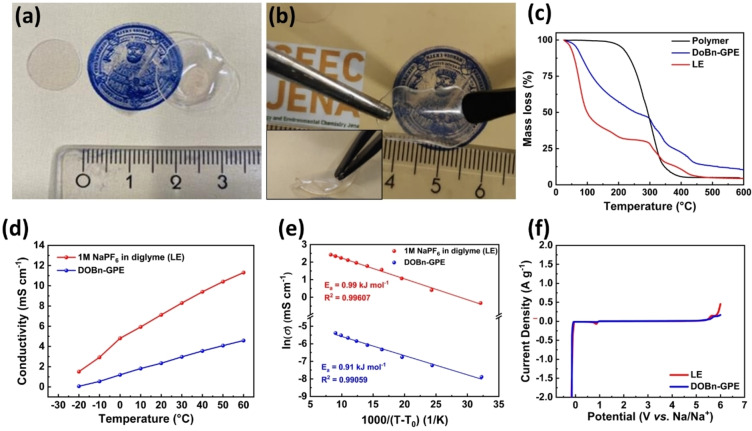
(a) Photograph of the polymer film (left) and the gellated DOBn‐GPE (right). (b) Mechanical properties of the DOBn‐GPE. (c) TGA of the polymer. (d) Temperature‐dependent conductivity measurements of the DOBn‐GPE compared to the LE. (e) Corresponding VTF‐plot. (f) Comparison of ESW of the GPE with LE determined by linear‐sweep voltammetry at a scan rate of 0.5 mV s^−1^.

The polymer matrix for the investigated GPE was synthesized via UV‐polymerization of two methacrylates (OEGMA and BnMA) with benzophenone as initiator (Scheme S1, Supporting Information).^[20a]^ The kinetic study revealed a polymerization time of 60 min with a monomer conversion of 99 % (Figure S1), and the resulting polymer film exhibited good mechanical properties (Figure S2). After gelation of the polymer film with the liquid electrolyte 1 m NaPF_6_ in diglyme (uptake 381 %) a freestanding GPE was obtained, which is referred to as DOBn‐GPE. DOBn‐GPE exhibited high mechanical stability (Figure 1a,b) and could be conveniently employed as a separator in an electrochemical cell. The thermal properties of the DOBn‐GPE were investigated by thermogravimetric analysis (TGA) and compared with those of the pure polymer film and the LE. As shown in Figure 1c and Figure S3a, DOBn‐GPE displayed a decomposition temperature (*T*_d_) of 64 °C, which is a lower value compared to that of the polymer matrix (210 °C), but higher than that of the LE (46 °C) that is limited by the relatively high vapor pressure of diglyme solvent. DOBn‐GPE revealed a glass transition temperature (*T*_g_) of −21 °C, whereas the polymer film showed a *T*_g_ of −2 °C. The ionic conductivity (*σ*) of both DOBn‐GPE and LE is, as expected, increasing with increasing temperatures (Figure 1d). At 20 °C LE displays a conductivity of 7.1 mS cm^−1^, while the conductivity of DOBn‐GPE is 2.3 mS cm^−1^. Although lower compared to that of the LE, it is important to notice that the conductivity value for DOBn‐GPE is outperforming those reported for many of the Na‐based GPEs considered so far (Table S1).[^17a^, ^21^] Both electrolytes follow the Vogel‐Tamman‐Fulcher (VTF) dependence of the ionic conductivity (Figure 1e), and the activation energy of DOBn‐GPE was higher than that of LE (0.91 vs. 0.99 kJ mol^−1^, respectively). The electrochemical stability window (ESW) of the DOBn‐GPE is comparable to that of the LE, and both are stable from 0 V vs. Na^+^/Na up to more than 5. 0 V vs. Na^+^/Na (Figure 1f). Considering the results reported above, the properties of DOBn‐GPE appear suitable for use in different types of Na‐based energy storage devices such as NICs, NMBs, and NIBs. In the following, the use of DOBn‐GPE in these devices will be considered in detail.

### DOBn‐GPE based Na‐ion capacitor

In the last years, several studies have been dedicated to the development of NIC.^[22]^ Most of these reports focused on the development of electrode materials,^[23]^ and only few studies considered innovative electrolytes.[^17c^, ^24^] Some of these latter investigations reported the use of diglyme‐based electrolytes in NICs[^10a^, ^10c^] but, to the best of our knowledge, no studies have been dedicated to diglyme‐based GPEs such as DOBn‐GPE.

**Figure 2:**
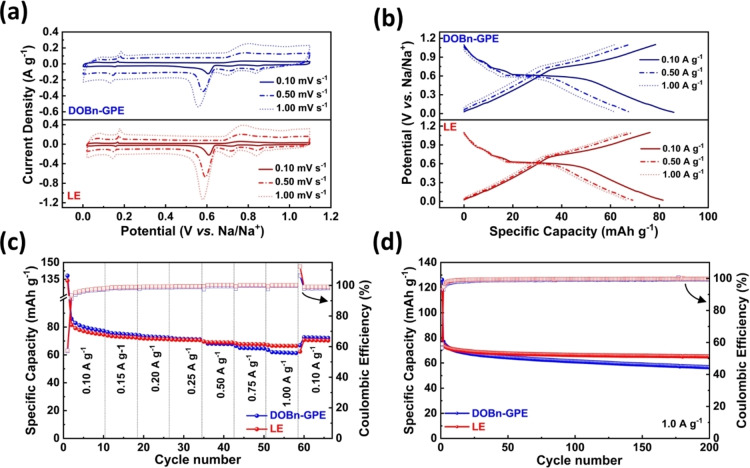
Electrochemical characterization of graphite electrode in DOBn‐GPE and LE: (a) CV obtained at different scan rates. (b) Voltage profile at different current densities. (c) Rate performance and (d) cycling stability of graphite in both electrolytes performed at a current density of 1.0 A g^−1^ (the Figure shows the discharge capacity).

As mentioned in the introduction, one of the main advantages associated with the use of diglyme‐based electrolyte is the possibility to realize Na‐based devices, including NICs, containing graphite electrodes. In order to realize such devices, however, it is essential to understand the influence of DOBn‐GPE on the Na‐ion co‐intercalation process in graphite electrodes. Figure 2a displays a comparison of the cyclic voltammetry (CV) of a graphite electrode in DOBn‐GPE and LE in the voltage range 0.0 to 1.1 V vs. Na^+^/Na at different scan rates (additional information available in Figure S4a). As shown in Figure 2a, at 0.1 mV s^−1^ the graphite electrodes revealed a similar behavior in LE and in DOBn‐GPE, and in both electrolytes the Na‐ion co‐intercalation is taking place at 0.6 V vs. Na/Na^+^. Interestingly, also when the scan rate is increased the behavior of the graphite electrodes in the two electrolytes remains comparable. The similar impact of the two electrolytes on the behavior of the graphite electrode is well visible also in Figure 2b,c, in which the voltage profiles, coulombic efficiency, capacity, and capacity retention of the electrodes are considered (see also S4b in the Supporting Information). When current densities equal to or lower than 0.5 A g^−1^ are applied, the efficiency, capacity, and capacity retention of the graphite electrode are almost identical in the two electrolytes. At higher current densities, the use of LE leads to slightly better performance values compared to that of DOBn‐GPE. At 1.0 A g^−1^, the graphite electrode cycled in combination with DOBn‐GPE and LE retained 72 and 82 %, respectively, of the capacities displayed at 0.10 A g^−1^. This difference of capacity at higher current rates can be ascribed to the resistance displayed by the polymer chains to the ionic movement, which is visible from the increased solution and charge transfer resistance determined from the fitted Nyquist plot (Figure S4c,e and Table S2). Nevertheless, it is interesting to observe that the graphite electrode cycled in DOBn‐GPE revealed a relaxation time constant $\left(\tau_{0}\right)$ of 12.5 s, which is close to that observed for the electrode cycled in LE (10 s) (Figure S4d and Table S2). It is also important to note that the use of DOBn‐GPE is not negatively affecting the electrode stability during 200 charge–discharge cycles carried out at 1.0 A g^−1^ (Figure 2d).

**Figure 3:**
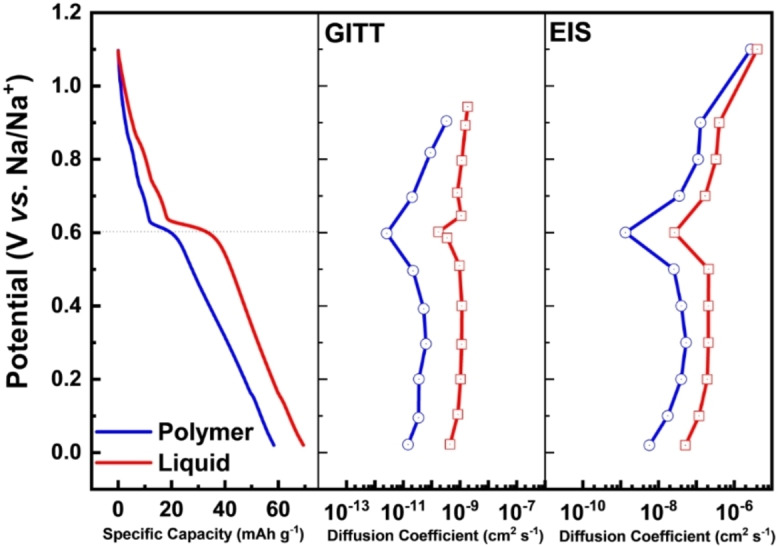
Diffusion coefficients of Na ions in graphite electrode during sodiation in the presence of DOBn‐GPE and LE, calculated from GITT and EIS measurements at different potentials.

To further understand the influence of DOBn‐GPE on the diffusion of solvated Na ions inside the graphite electrodes, the diffusion coefficient at different intercalation potentials was determined by employing galvanostatic intermittent titration technique (GITT) and electrochemical impedance spectroscopy (EIS) (Figure S5). Figure 3 displays the variation of the diffusion coefficient of Na ions within the graphite in DOBn‐GPE and in LE at different potentials. As shown, the diffusion coefficient of Na ions is following an identical trend through all the considered potential range although the ions are diffusing faster in LE than in DOBn‐GPE. This difference might be caused by a different electrode wetting and a different Na‐ion solvation in the two electrolytes. It is important to point out, nonetheless, that the Na ions are diffusing relatively fast in DOBn‐GPE although this is a GPE. As shown, the diffusion coefficient shows a minimum value at 0.6 V, where maximum co‐intercalation is taking place.

**Figure 4:**
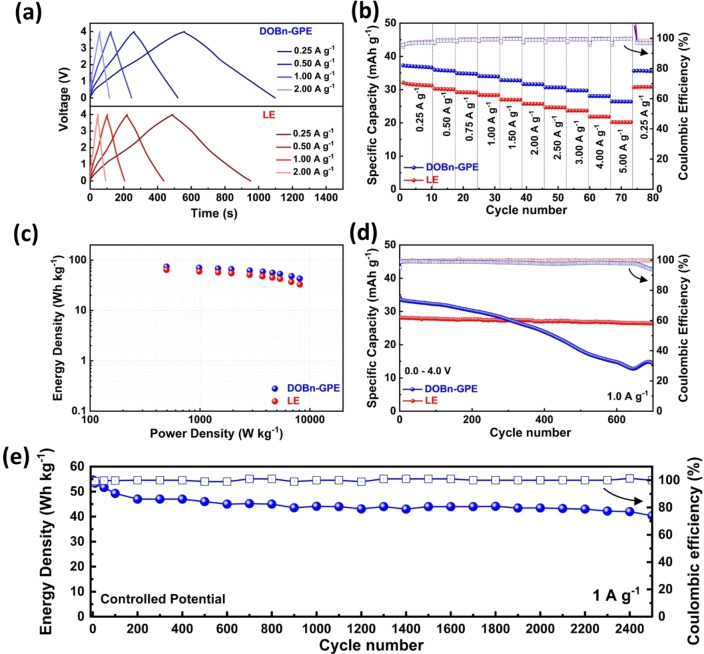
Electrochemical performance of NICs containing DOBn‐GPE and LE as electrolyte: (a) GCD curve. (b) Rate capability of at different current densities in a voltage window of 0.0–4.0 V. (c) Ragone plot and (d) stability measurements of both devices at 1.0 A g^−1^ in a voltage window of 0.0–4.0 V. (e) Long‐term cycling stability of graphite electrodes in DOBn‐GPE measured at a current rate of 1.0 A g^−1^ in a controlled anode potential method.

After the investigations discussed above, the graphite electrodes were utilized in combination with an AC‐based electrode for the realization of NIC containing DOBn‐GPE and LE as electrolytes, and operating at 4 V. All the details about the NIC are available in the Experimental Section. Figure 4a and Figure S6a compare the GCD profile of NICs in the DOBn‐GPE and LE at 1 A g^−1^. As shown, both devices revealed the typical profile (triangular shape) of NICs. Figure 4b compares the capacity retention of the devices during tests carried out from 0.25 to 5.00 A g^−1^ (based on the active mass of both electrodes). As shown, at 0.25 A g^−1^ the DOBn‐GPE‐based NIC revealed a capacity of 37 mAh g^−1^, while that of the LE‐based NIC is 32 mAh g^−1^, indicating that the use of the investigated electrolytes leads to NICs with similar performance. Furthermore, it is very interesting to observe that the two NICs displayed also a very comparable capacity retention (≈97 %) through all the considered current densities. At 5 A g^−1^ the NIC containing DOBn‐GPE revealed a capacity of 26 mAh g^−1^, while the LE‐based NIC had a capacity of 20 mAh g^−1^, which are corresponding to a decrease of 30 and 38 %, respectively, compared to the initial value. As shown in the Figure 4, both devices displayed high coulombic efficiency during the charge–discharge tests, and both of them are able to fully recover their initial capacity when a current density of 0.25 A g^−1^ is applied. Moreover, the DOBn‐GPE‐based NIC showed only a slight rise in the charge transfer resistance (from 3.1 to 4.6 Ω) and the equivalent series resistance (from 25.6 to 29.0 Ω) after the rate capability measurements (Table S3). Additionally, the relaxation time constant ($\tau_{0}$ ) of this device increased only slightly over the time of the test (from 6.7 s to 7.1 s; see Figure S6c,d and Table S3). The similar behavior of the two devices is also well reflected in the Ragone plot shown in Figure 4c, where both devices exhibited comparable energy and power. It is important to outline that the DOBn‐GPE based NIC at a power density of 8 k W kg^−1^ displayed an energy density of 43 Wh kg^−1^, which is one of the best performances reported so far for NICs containing GPEs. Figure 4d compares the cycling stability of the two investigated NICs. As shown, the stability of the NIC containing LE is higher than that of the system containing DOBn‐GPE, and after 500 cycles this latter device lost 50 % of its initial capacity. This lower stability is mainly caused by an undesirable electrode drift occurring on the negative electrode of the device.^[3f]^ As shown in Figure 4e, the stability of the device can be significantly improved by controlling (and limiting) the behavior of the negative electrode. Utilizing this strategy, the device is able to retain approximately 75 % of its initial energy density after 2500 cycles. This latter strategy is clearly not suitable for practical application, and the optimization of a two‐electrode device is certainly needed. Nonetheless, these results indicate that the new DOBn‐GPE is a suitable electrolyte for the realization of high‐performance NICs.

### DOBn‐GPE based Na‐metal and Na‐ion batteries

The realization of NMBs represents a topic of great interest, as these devices display very high energy density. Nevertheless, as in the case of lithium‐metal batteries, their development is challenging due to the high reactivity of the metal electrodes, which limits the safety and cycle life of the devices.[^12c^, ^12d^, ^25^] Seh et al.^[11]^ reported that the utilization of the LE considered in this work (1 m NaPF_6_ in diglyme) mitigates the dendrite formation at the Na‐metal electrode, enabling the realization of NMBs with good cycling stability. Thus, to consider the use of DOBn‐GPE in NMBs appears of high interest because a successful introduction of this GPE in these devices could improve their safety and limit the risk of leakage during their use.

**Figure 5:**
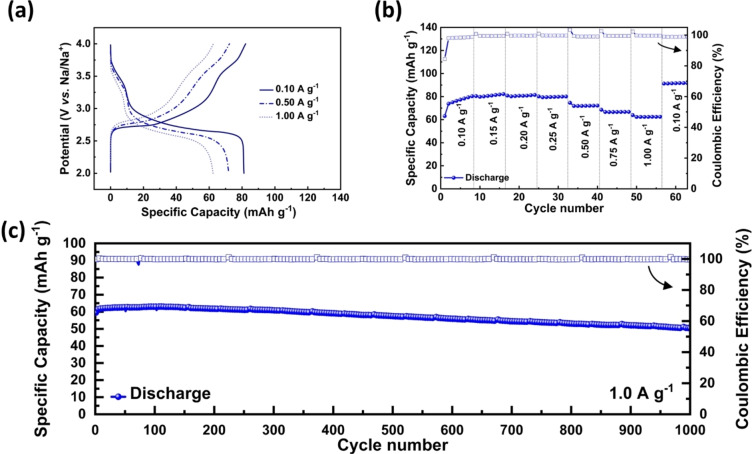
Electrochemical performance of DOBn‐GPE‐based NMB at room temperature: (a) GCD. (b) Rate performance at different current densities. (c) Long‐term cycling performance at a current density of 1.0 A g^−1^.

To investigate this aspect, we utilized DOBn‐GPE in combination with a PB cathode and a Na‐metal anode. All the details about the cell are available in the Experimental Section. Figure 5a and Figure S7a show the charge–discharge curves at different current densities of the investigated cell, which is cycled between 2.0–4.0 V at room temperature (the CVs of the cells are available in Figure S7b). As shown, the cell displays the typical voltage profile of PB‐based systems,^[26]^ and at a current density of 0.1 A g^−1^ (which corresponds to ≈0.8 C considering 124.8 mA g^−1^ as the theoretical capacity of PB) it delivers a specific capacity of 81 mAh g^−1^. When the current density is increased, the cell retains very well its capacity and at 1.0 A g^−1^ displays a specific capacity of 62 mAh g^−1^ with an average coulombic efficiency of 99.6 % (Figure 5b). Interestingly, when the current density is reduced again to 0.1 A g^−1^ the cell displays a capacity 92 mAh g^−1^, which is a higher value than that observed at the beginning of the test. The high capacity and the good rate performance enabled the achievement of very promising values of energy and power for these devices (≈168 Wh kg^−1^ and 2.7 kW kg^−1^ at 1.0 A g^−1^; see Ragone plot in Figure S7c). It is important to point out that the cell also displays excellent cycling stability and is able to retain 85 % of its initial capacity after 1000 charge‐discharge cycles carried out at a current density of 1.0 A g^−1^ (Figure 5c).

**Figure 6:**
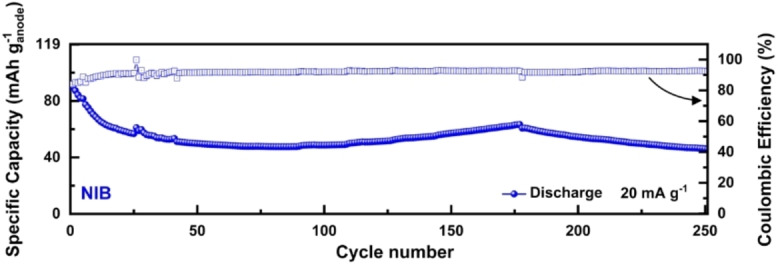
Cycling stability of DOBn‐GPE‐based NIB at room temperature obtained at 20 mA g^−1^.

The results discussed above indicate that the new DOBn‐GPE can be successfully utilized in combination with graphite and PB electrode. As a consequence, we combined these two electrode materials to investigate the use of DOBn‐GPE in NIBs. All the details about the cell are reported in the Experimental Section. It has to be mentioned that the goal of this study is not to realize an optimized cell, but rather to understand the impact of DOBn‐GPE on the cycling behavior of lab‐scale NIBs. As shown in Figure 6, after an initial capacity decay during the first 20 cycles, which was most likely associated with an imperfect electrode balancing, the cell behavior stabilized, and after 250 cycles the cell retained around 52 % of the initial capacity. Further optimizations appear necessary to reduce the initial capacity fade of the cell, but, overall, the use of DOBn‐GPE in NIB appears promising.

### DOBn‐GPE based devices operating at 60 °C

**Figure 7:**
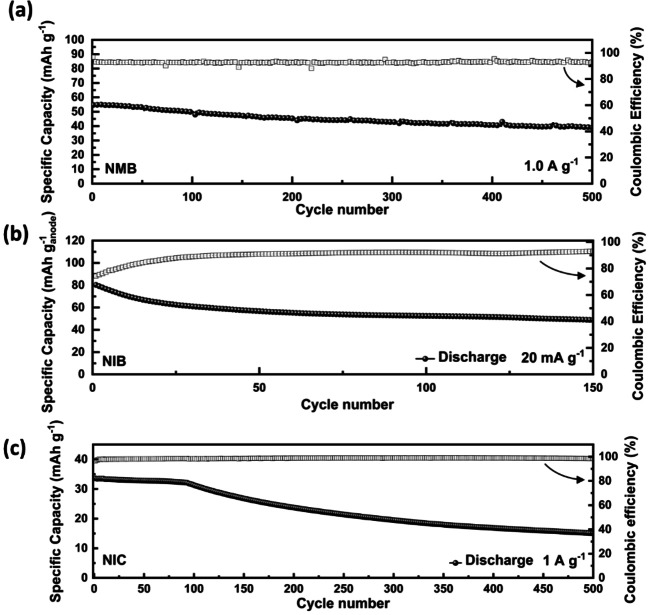
Cycling stability of: (a) NIB, (b) NMB, and (c) NIC containing DOBn‐GPE at 60 °C.

The behavior of the devices discussed in the previous section indicates that DOBn‐GPE can be considered as a promising electrolyte for sodium‐based devices operating at room temperature. Since it is well known that in many application the use of higher temperature is required,^[27]^ we also carried out a preliminary study about the cycling stability of the DOBn‐GPE based devices at 60 °C. Figure 7 displays the variation of the specific capacity over the cycle number of the NMB, NIB, and NIC considered above at this temperature. As shown in Figure 7a, the NMB displays at 60 °C an initial capacity of 55 mAh g^−1^, comparable to that observed at room temperature (more details about the capacity retention are available in Figure S8a). The device displays promising energy and power (≈179 Wh kg^−1^ at 2.8 kW kg^−1^, see Figure S8b) and after 500 cycles carried out at 1 A g^−1^ is able to retain more than 71 % of its initial capacity. This latter result indicated that the new DOBn‐GPE is able to assure high stability and displays very good compatibility with metallic sodium also at high temperature. Figure 7b shows the stability at 60 °C of the NIB. At this temperature the device displays an initial specific capacity of 80 mAh g^−1^, comparable to the capacity measured at room temperature. After 150 cycles a specific capacity of 50 mAh g^−1^ could be measured, which correspond to a capacity retention of 63 %. Considering the fact that the cell was not optimized (see comment above), this result appears certainly very promising. Finally, also the stability at 60 °C of the NIC has been investigated. As shown in Figure 7c, the NIC cycled in a potential controlled method at 1.0 A g^−1^ at 60 °C display an initial capacity of 35 mAh g^−1^, which is a promising value for this kind of device. After 500 cycles the device was able to retain half of its initial capacity. This latter value indicates that the stability of the devices is affected by the temperature, most likely because the electrodes during the cycling are not working in an optimal voltage range, accelerating the occurrence of degradation processes.^[20c]^

## Conclusion

In this study, we reported for the first time a new diglyme‐based gel polymer electrolyte (DOBn‐GPE) suitable for Na‐based energy storage devices. The DOBn‐GPE, which is based on a methacrylate‐based polymer, exhibited high ionic conductivity (2.3 mS cm^−1^ at 20 °C), broad electrochemical stability (>5.0 V), and is highly mechanically stable. We demonstrated that DOBn‐GPE can be successfully utilized in sodium‐ion capacitors (NICs), sodium‐metal batteries (NMBs), and sodium‐ion batteries (NIBs). The NIC fabricated with DOBn‐GPE exhibited performance values comparable to the device containing the (same) liquid electrolyte, and it displayed an energy density of 43 Wh kg^−1^ at the very high power density of 8 k W kg^−1^. The NMB containing DOBn‐GPE displayed high capacity (62 mAh g^−1^ at 1.0 A g^−1^) and high cycling stability (85 % capacity retention after 1000 cycles), indicating that DOBn‐GPE can be successfully used also with a Na‐metal anode. The use of DOBn‐GPE in NIB allowed the achievement of a lab‐scale cell with promising results (52 % of capacity retained after 250 cycles). Finally, we demonstrated that the new DOBn‐GPE can be successfully utilized at 60 °C, and that at this high temperature Na‐based devices containing this electrolyte displayed high capacity and high cycling stability.

The results of these investigations are indicating that the development of diglyme‐based GPEs represents a promising strategy for the realization of advanced Na‐based energy storage devices. Future work will focus on better understanding the Na‐ion coordination within the electrolytes and, in addition, realizing even more optimized polymers especially designed for use in combination with ether‐based solvents.

## Experimental Section

### Preparation and characterization of DOBn‐GPE

The polymerization process of the polymer matrix has been adapted from a procedure already reported in the literature.^[20a]^ All chemicals used were purchased from Merck and Aldrich. The monomers were passed over a short neutral aluminum oxide plug before use.

In order to obtain the mixture for preparation of the polymer matrix 286 μL (300 mg, 1 mmol) oligo(ethylene glycol) methyl ether methacrylate (OEGMA) and 508 μL (528.65 mg, 3 mmol) benzyl methacrylate (BnMA) were mixed with 1.66 mg (0.2 wt%) benzophenone. The formulation was placed between two siliconized polyethylene terephthalate (PET) foils (Figure S1) and polymerized for 1 h by UV‐irradiation (film thickness was adjusted with a spacer of 200 μm). Afterward, disks were punched out of the polymer film and dried overnight in a Büchi oven at 120 °C. Subsequently, the polymer films were soaked overnight under an inert atmosphere with 1 m NaPF_6_ in diglyme (polymer/LE=1 : 4).

1D (^1^H) NMR spectra were recorded on a Bruker AC 300 (300 MHz) at 298 K. Chemical shifts are reported in parts per million (ppm, *δ* scale) relative to the residual signal of the solvent. TGA measurements (under helium) were carried out on the polymer using a STA Netzsch 449 F3 Jupiter. Differential scanning calorimetry (DSC) measurements were carried out with a DSC 204 F1 Phoenix by Netzsch under a nitrogen atmosphere with a heating rate of 10 or 20 K min^−1^.

### Electrochemical characterization of DOBn‐GPE and LE

The ionic conductivity of DOBn‐GPE was measured a closed cell (TSC Battery cell from rhd instruments) between two stainless‐steel electrodes. Temperature‐dependent conductivity measurements were performed in a Binder “Alternating Climate Chamber MK056”. For LE, the samples were placed in sealed cells with Pt‐plated electrodes. All the conductivities were determined via impedance measurements using a potentiostat ModuLabXM (Solartron analytical). The measurements were carried out in the frequency range of 300 kHz to 1 Hz with an alternating current of 5 mV to analyze the resistance with a in a temperature range between −20 and 60 °C.

The ESW of both DOBn‐GPE and LE was evaluated by linear sweep voltammetry (LSV) in a three‐electrode configuration cell. The working electrode (WE) consisting of stainless steel (area 1.13 cm^2^, diameter 12 mm) and metallic sodium was used as the counter and reference electrode. For LE, a Whatman GF/D glass microfiber filter (675 μm thickness and 13 mm diameter) drenched with 150 μL of 1 m NaPF_6_ in diglyme electrolyte was used as the separator. The anodic and cathodic limit of both electrolytes was measured by sweeping the potential of the WE at a scan rate of 0.5 mV s^−1^ in a more positive and more negative potential range.

### Electrode preparation and device fabrication

The graphite electrodes were prepared by mixing commercial graphite (SFG 6, Imerys, Switzerland) with a conductive carbon (Super C 65, Imerys, Switzerland) and a binder (carboxy‐methyl cellulose, CMC, Walocel CRT 2000 PA, Dow Wolff Cellulosics, German) in a mass ratio of 90 : 5 : 5 in ultra‐pure distilled water solution following a procedure similar to that described previously in the literature.^[28]^ The activated carbon electrodes were prepared utilizing a similar procedure, but using Super 30‐DLC Super (Norit) as the active material. The graphite electrodes were coated on copper foil, whereas the activated carbon electrodes were coated on aluminum foil. All electrodes were dried at 100 °C for at least 12 h under vacuum conditions. All electrodes had an area of 1.13 cm^2^ and an active mass loading between 1–2 mg cm^−2^.

The PB electrode were prepared utilizing commercial iron(III)hexacyanoferrate(II) (Alfa Aesar) as Super C 65 and CMC as the conducting agent and binder, respectively. The mass ratio active material/conducting agent/binder was 80 : 10 : 10. The slurry was coated on aluminum foil and was kept at 100 °C for at least 12 h under vacuum for drying. The punched electrodes had an area of 1.13 cm^2^ and an active mass loading of around 4.5 mg cm^2^. A Na‐metal foil disk (12 mm diameter) was used as the counter electrode for the tests in half cell.

All the investigated cells were fabricated using a Swagelok®‐type cell, assembled in an argon‐filled glovebox (MBraun, <1 ppm H_2_O and <1 ppm O_2_). The NICs were realized with a pre‐sodiated graphite electrode (negative electrode) and a pre‐cycled activated carbon electrode (AC, positive electrode) along with the DOBn‐GPE and LE. For LE, a Whatman GF/D glass microfiber filter (675 μm thickness and 13 mm diameter) drenched with 150 μL of 1 m NaPF_6_ in diglyme electrolyte was used as the separator. The NICs was fabricated utilizing an active mass ratio equal of 1 : 1 between positive and negative electrode in order to avoid the polarization at the graphite electrode, which improves the cycling stability of the device.^[3c]^ Before being utilized in the NICs, the graphite electrode was pre‐sodiated following a protocol identical to that reported in a previous work of our group.^[20c]^

For the fabrication of NMBs, the PB electrode was utilized as cathode, and the Na‐metal foil was used as anode. DOBn‐GPE was used as electrolyte‐separator. The NIBs were fabricated with pre‐sodiated graphite as anode and pre‐cycled PB as the cathode in an electrode mass ratio (anode/cathode=1 : 3), and DOBn‐GPE was used as electrolyte‐separator.

### Electrochemical characterization of devices

All the electrochemical characterizations such as GCD, CV, GITT, and EIS measurements have been carried out using VMP3 (BioLogic) and LBT21084 (Arbin Instruments). All the Nyquist plots were fitted by using the Z‐Fit technique in EC‐Lab software with low $\chi^{2}$ ‐value.

The electrochemical characterization of graphite electrode in DOBn‐GPE and LE was conducted in a two‐electrode configuration with metallic sodium as counter and reference electrode. GITT and EIS measurements were conducted to analyze the Na‐ion diffusion coefficient in graphite electrodes in the presence of DOBn‐GPE and LE.

The investigated NICs were characterized in a three‐electrode cell Swagelok®‐type cell setup and contained a graphite‐based negative electrode, an activated carbon‐based positive electrode, and metallic sodium as the reference electrode. DOBn‐GPE and LE were used as electrolytes. The NICs were cycled in a potential window of 0–4 V. The long‐term cycling stability measurement of NICs containing DOBn‐GPE was also measured in a potential‐controlled method by limiting the anode potential in the range of 0–1.5 V. In the case of NMBs, all the electrochemical characterizations were conducted in a two‐electrodes configuration in the potential window of 2.0–4.0 V. The thickness of Na‐metal foil was between 150–200 μm. Further, the long‐term cyclic stability of NIBs was characterized in a three‐electrode configuration with metallic sodium as the counter electrode and measured in a potential‐controlled method. All devices (NMBs, NIBs, and NICs) were investigated at room temperature and at 60 °C.

The diffusion coefficient of Na ions in graphite electrode was estimated utilizing GITT and EIS. During the GITT measurements, the graphite electrode was perturbed (sodiated/de‐sodiated) with a current flux density of 20.0 mA g^−1^ for a transient time of 300 s and provided a resting time of 1.0 h. The obtained voltage vs. time profile is shown in Figure S5a,b. The chemical coefficient ($D_{chem}$ ) of Na ions inside the graphite at different depth of discharge (DOD) and state of charge (SOC) was determined utilizing Equation (1), according to Fick's second law of diffusion:^[29]^

(1) $$D_{chem}=\;\frac{4}{\pi t}\;{\left(\frac{m_{B}V_{M}}{M_{B}A}\right)}^{2}{\left(\frac{\Delta E_{s}}{\Delta E_{\tau }\;}\right)}^{2}$$

where $m_{B}$ , $M_{B}$ , and $V_{M}$ are the mass loading [g], molar mass [g mol^−1^], and molar volume [cm^3^ mol^−1^] of the active host material, respectively. A is the contact area between the electrolyte and the active material. Here we used the geometrical surface area of the electrode. ▵$E_{\tau }$ is the potential perturbed during the transient time and ▵$E_{s}$ is the difference between the two adjacent quasi‐equilibrium potentials attained after the relaxation time.

During the EIS measurements, the graphite electrodes were subjected to an AC signal of amplitude 5 mV in a frequency range of 500 kHz to 1.0 mHz at different open circuit voltages (OCVs) during sodiation and de‐sodiation. The frequency responses of the system were analyzed and presented in a complex plane by cole‐cole or Nyquist plots, as shown in Figure S5c,d.

The Na‐ion diffusion coefficient in graphite at different DOD and SOC were determined from the EIS experiments by using Equation (2), according to Fick's second law of diffusion:^[30]^

(2) $$D_{chem}=\;\frac{1}{2}{\left[\left(\frac{V_{M}}{\sigma_{w}AFz}\right)\left(\frac{\delta E}{\delta x}\right)\right]}^{2}$$

where F is the Faraday constant and $\sigma_{w}$ is the Warburg co‐efficient or co‐factor, which is obtained from the average value of the slope of $Z{}^{'}$ vs.${\;\omega }^{-\frac{1}{2}}$ and -Z" vs.${\;\omega }^{-\frac{1}{2}}$ .

## Conflict of interest

The authors declare no conflict of interest.

## Supporting information

As a service to our authors and readers, this journal provides supporting information supplied by the authors. Such materials are peer reviewed and may be re‐organized for online delivery, but are not copy‐edited or typeset. Technical support issues arising from supporting information (other than missing files) should be addressed to the authors.

Supporting Information
