# Supplementary material for: New Diglyme‐based Gel Polymer Electrolytes for Na‐based Energy Storage Devices
Source: ChemSusChem. 2021 Sep 30;14(21):4836–45. doi: 10.1002/cssc.202101445 (PMC8597054; doi:10.1002/cssc.202101445)
Supplement: Supplementary file 1 — Supporting Information [file CSSC-14-4836-s001.pdf]

ChemSusChem

Supporting Information

New Diglyme-based Gel Polymer Electrolytes for Na-based Energy Storage Devices

Binson Babu, Marcel Enke, Sofiia Prykhodska, Alexandra Lex-Balducci, Ulrich S. Schubert, and Andrea Balducci*© 2021 The Authors. ChemSusChem published by Wiley-VCH GmbH. This is an open access article under the terms of the Creative Commons Attribution License, which permits use, distribution and reproduction in any medium, provided the original work is properly cited.

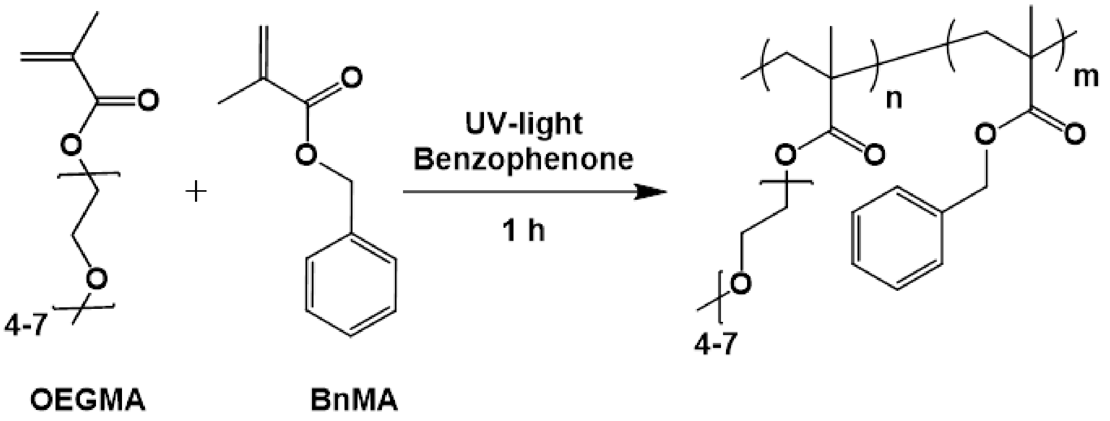

Scheme S1. Schematic representation of the free radical polymerization of oligo(ethylene glycol) methyl ether methacrylate (OEGMA) and benzyl methacrylate (BnMA).

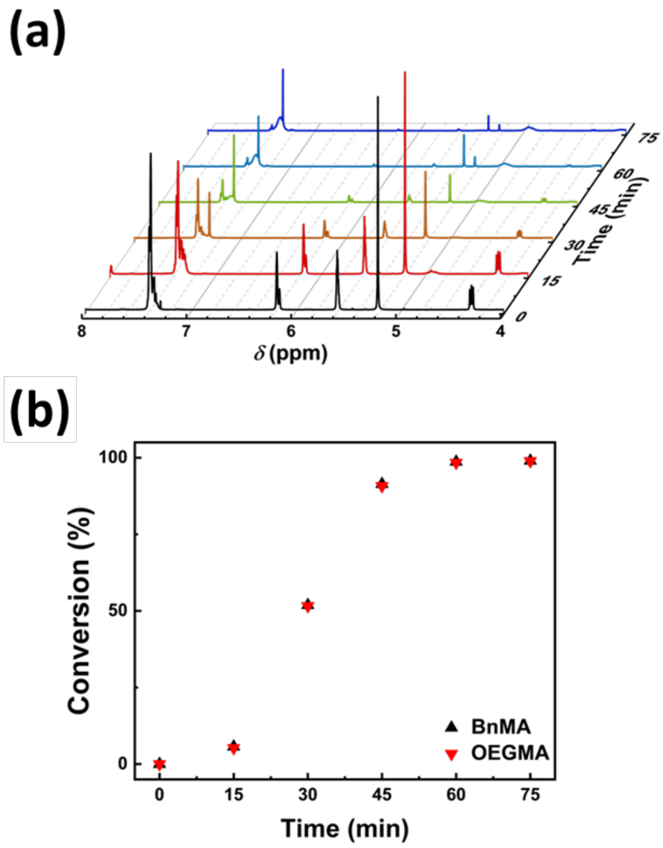

Figure S1. (a) Proton-NMR kinetics of the copolymerization of OEGMA and BnMA (monomer signals in between 5.0 to 6.5 ppm) with benzophenone under UV-irradiation and (b) time evolution of monomer conversion which shows a 99% conversion after 60 min.

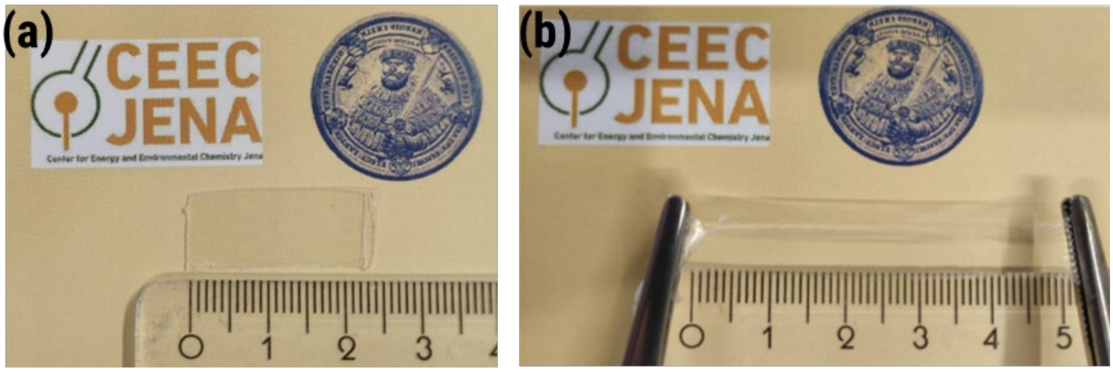

Figure S2. Mechanical properties of the polymer film: (a) Photograph of the prepared polymer film before stretching and (b) stretching of the pure polymer up to 120% strain.

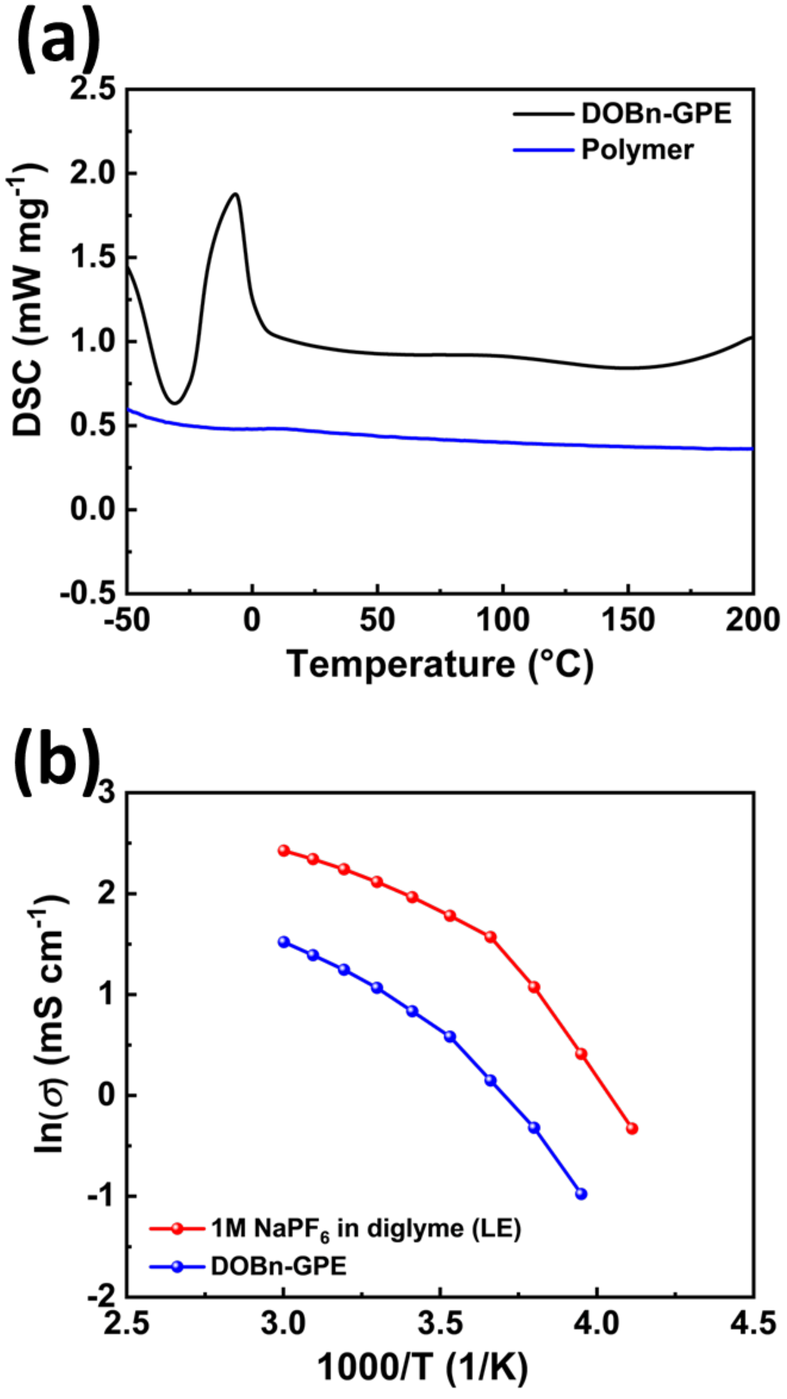

Figure S3. (a) Differential scanning calorimetry (DSC) of the polymer (T_g = −2 °C) and the gel-polymer electrolyte (T_g = −21 °C). (b) Arrhenius plot of the temperature-dependent conductivity measurements of the DOBn-GPE compared to the LE.

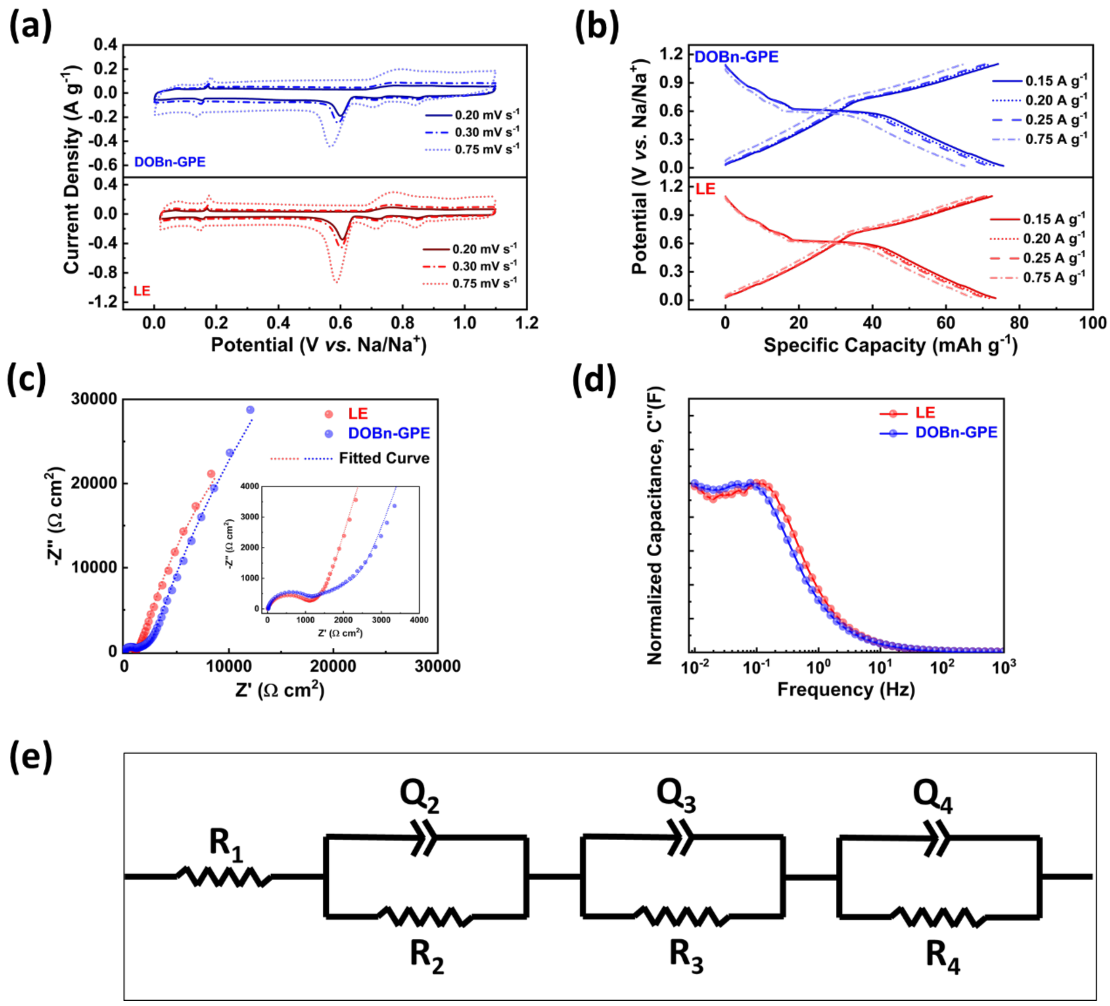

Figure S4. a) Voltage profile at different current densities and b) cyclic voltammetry at different scan rates of graphite *vs.* Na-Metal in DOBn-GPE and LE. c) Nyquist plots along with fitted curve compares the impedance performance of graphite *vs.* Na-metal in DOBn-GPE and LE d) shows the normalized imaginary capacitance *vs.* Frequency plots derived from EIS measurements. e) Equivalent circuit used to fit the Nyquist curves shown in c)

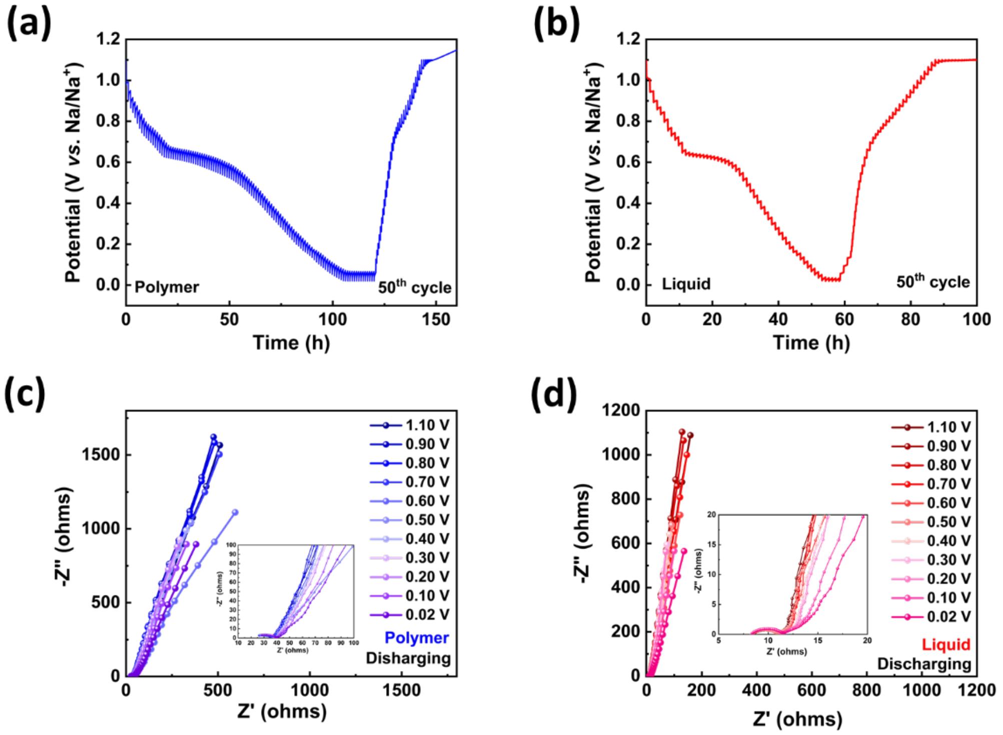

Figure S5. Voltage profile of graphite *vs.* Na-Metal during GITT measurement in a) DOBn-GPE and b) LE. c) and d) represents the Nyquist plot at different Na-ion intercalation potentials in graphite electrodes in DOBn-GPE and LE, respectively. Inset shows the zoomed view of the Nyquist plot.

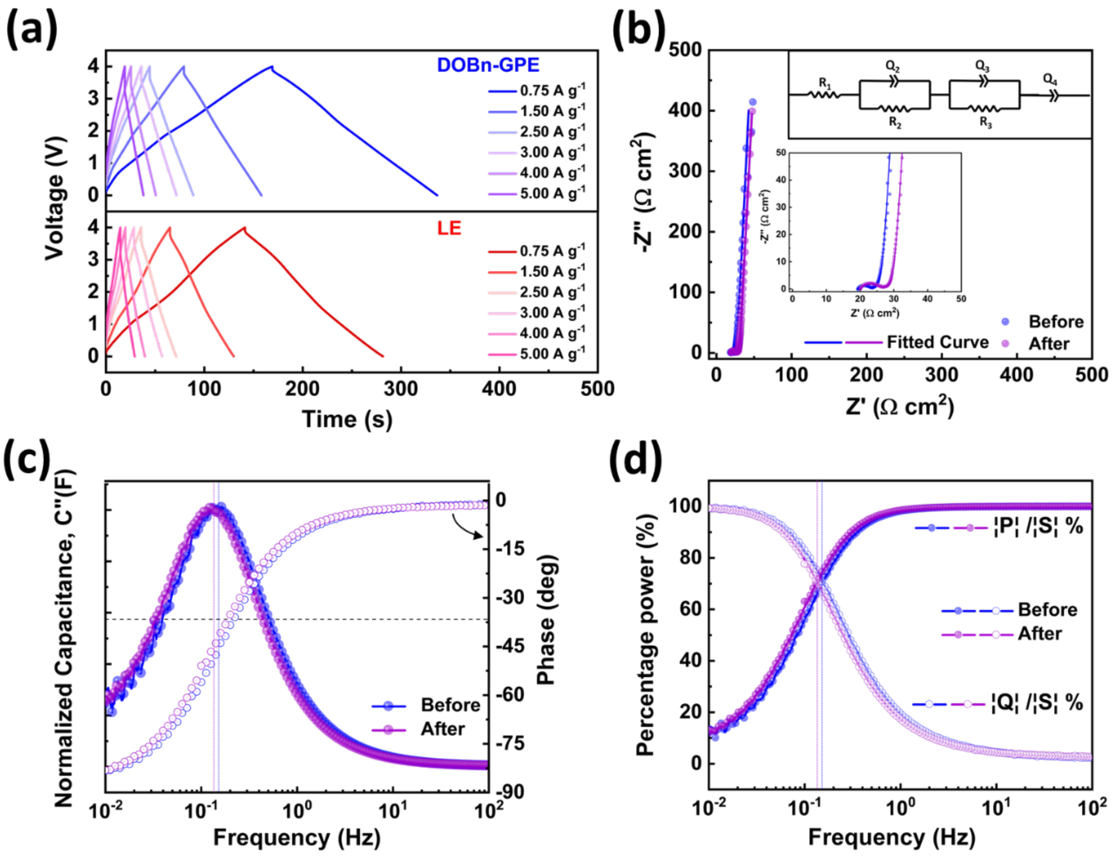

Figure S6. a) Galvanostatic charge/discharge profile of NIC containing DOBn-GPE and LE. b) Nyquist plots with the fitted curve of NIC containing DOBn-GPE‖AC before and after rate capability measurements (inset shows the equivalent circuit used for fitting). c) Evolution of normalized imaginary capacitance and phase angle *vs*. frequency and d) Normalized active power ¦P¦ /¦S¦ and reactive power ¦Q¦ /¦S¦ vs. frequency derived from EIS measurements of NIC containing DOBn-GPE.

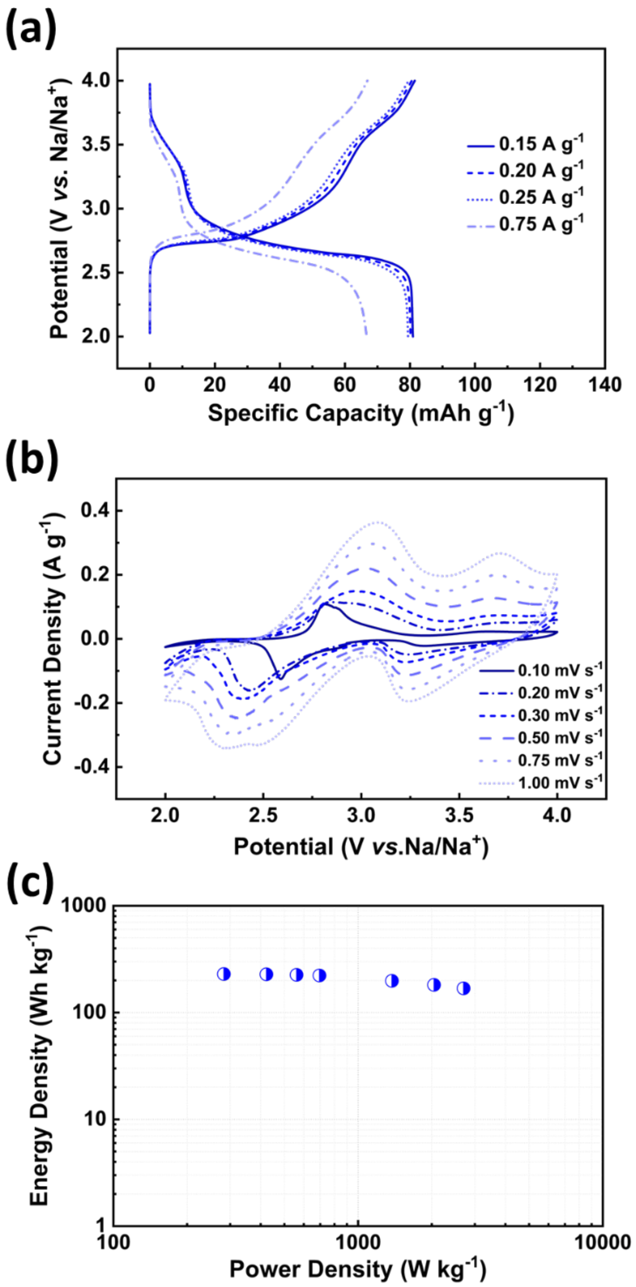

Figure S7. Electrochemical performance of DOBn-GPE-based NMB at room temperature: a) Voltage profile at different current densities, b) cyclic voltammetry at various scan rates of room temperature DOBn-GPE-based NMB. c) Ragone plot portrays the energy density *vs.* power density of DOBn-GPE-based NMB at different current rates (based on PB mass in cathode).

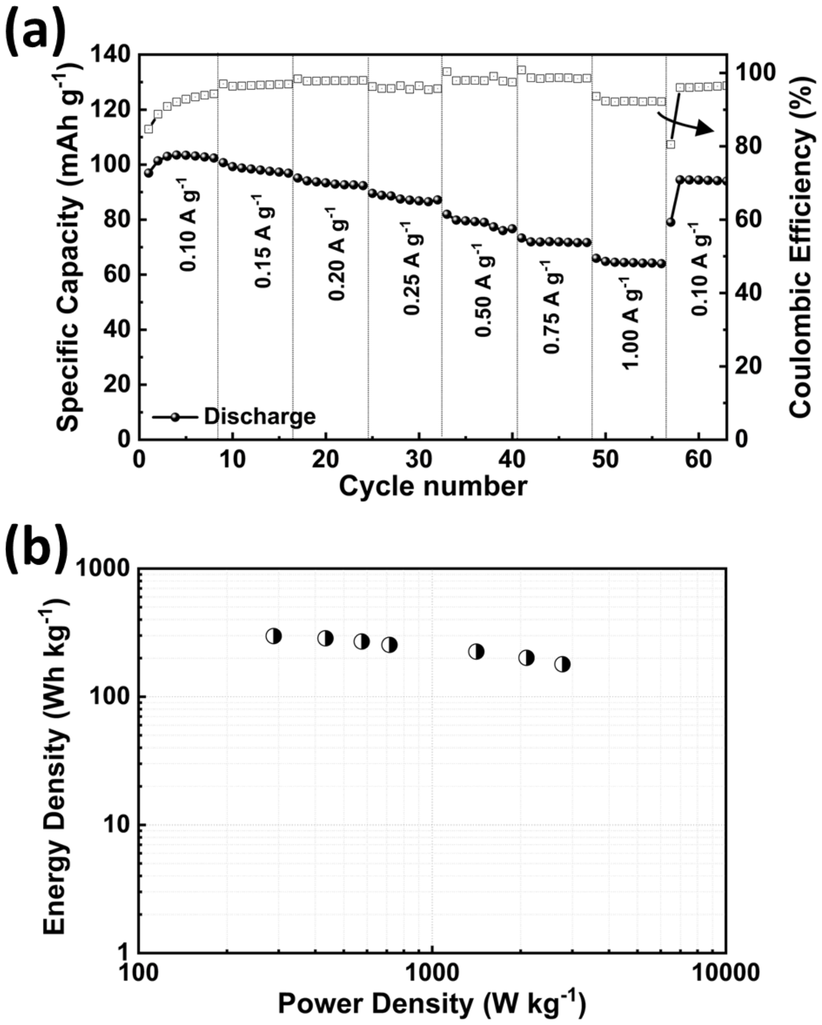

Figure S8. Electrochemical performance of DOBn-GPE-based NMB at 60 °C. a) Rate performance and b) Ragone plot portrays the energy density *vs.* power density (based on PB mass in cathode) of DOBn-GPE-based NMB at different current rates.

Table S1. The ionic conductivities and electrochemical stability windows of the representative polymer electrolytes

Type	Electrolyte		Ionic Conductivity ($S\ cm^{-1}$)	Electrochemical Stability Window (V)	Temp.	Refs.
GPE	ANs–PVdF–HFP		7.13 × 10−4 S cm^{-1}	4.8 V vs. Na/Na^{+} (1.0 mV s^{-1})	25	[1]
GPE	PU-$NaClO_4$-EC/PC/DEC		1.5 mS cm^{-1}	4.6 V vs. Na/Na^{+} (1.0 mV s^{-1})	25	[2]
GPE	ETPTA/HMPP-NaClO_4-PC/FEC		1.2 mS cm^{-1}	4.7 V vs. Na/Na^{+} (1.0 mV s^{-1})	RT	[3]
GPE	PPEGMA/ TEP/ FEC (PGT32-5%)		0.91 mS cm^{-1}	4.8 V vs. Na/Na^{+} (0.1 mV s^{-1})	RT	[4]
GPE	porous P(VDF-HFP) $NaClO_4$/EC/DMC/DEC		0.60 mS cm^{-1}	4.6 V vs. Na/Na^{+} (2.0 mV s^{-1})	RT	[5]
GPE	DOBn-GPE		2.3 mS cm^{-1}	> 5.0 V vs. Na/Na^{+} (0.5 mV s^{-1})	RT	This work

The abbreviations are listed as below: AN - β/β″-Al_2O_3 Nanowires, PVdF–HFP - poly(vinylidene fluoride-*co*-hexafluoropropylene), PU - Polyurethane, ETPTA - Ethoxylated trimethylolpropane triacrylate, PPEGMA - poly(ethylene glycol methyl ether methacrylate), TEP - triethyl phosphate, FEC -fluoroethylene carbonate, EC - Ethylene carbonate, DMC - Dimethyl carbonate, DEC - Diethyl carbonate.

Table S2. Physical parameters of Graphite *vs*. Na-metal half-cell configuration at OCV.

Electrolyte	Solution Resistance (Ω)	Charge transfer resistance (Ω)	Characteristic frequency, f_0 (Hz)	Relaxation time constant, $\tau_0 = \frac{1}{f_0}$ (s)
LE	7.7	874.1	0.1	10
DOBn-GPE	17.63	909.1	0.08	12.5

Table S3. Physical parameters derived from the EIS measurements of NIC in DOBn-GPE before and after the rate capability test.

NIC	Solution Resistance (Ω)	Charge transfer resistance (Ω)	ESR (Ω)	Characteristic frequency, f_0 (Hz)	Relaxation time constant, $\tau_0 = \frac{1}{f_0}$ (s)
Before	17.1	3.1	25.6	0.15	6.7
After	17.5	4.6	29.0	0.14	7.1

References

[1] Y. Luo, L. Liu, K. Lei, J. Shi, G. Xu, F. Li, J. Chen, *Chem. Sci.,* **2019**, *10*, 2048-2052.

[2] M.-S. Park, H.-S. Woo, J.-M. Heo, J.-M. Kim, R. Thangavel, Y.-S. Lee, D.-W. Kim, *ChemSusChem* **2019**, *12*, 4645-4654.

[3] P. Wen, P. Lu, X. Shi, Y. Yao, H. Shi, H. Liu, Y. Yu, Z.-S. Wu, *Adv. Energy Mater.* **2021**, *11*, 2002930.

[4] G. Chen, K. Zhang, Y. Liu, L. Ye, Y. Gao, W. Lin, H. Xu, X. Wang, Y. Bai, C. Wu, *Chem. Eng. Technol.* **2020**, *401*, 126065.

[5] Y. Q. Yang, Z. Chang, M. X. Li, X. W. Wang, Y. P. Wu, *Solid State Ion.* **2015**, *269*, 1-7.
